# US Nationwide Disclosure of Industry Payments and Public Trust in Physicians

**DOI:** 10.1001/jamanetworkopen.2019.1947

**Published:** 2019-04-12

**Authors:** Genevieve P. Kanter, Daniel Carpenter, Lisa S. Lehmann, Michelle M. Mello

**Affiliations:** 1 Division of General Internal Medicine, Department of Medicine, Perelman School of Medicine, University of Pennsylvania, Philadelphia; 2 Department of Medical Ethics and Health Policy, Perelman School of Medicine, University of Pennsylvania, Philadelphia; 3 Leonard Davis Institute of Health Economics, University of Pennsylvania, Philadelphia; 4 Radcliffe Institute for Advanced Study, Harvard University, Cambridge, Massachusetts; 5 Department of Government, Harvard University, Cambridge, Massachusetts; 6 National Center for Ethics in Health Care, Veterans Health Administration, Washington, DC; 7 Stanford Law School, Stanford University, Stanford, California; 8 Department of Health Research and Policy, Stanford University School of Medicine, Stanford, California

## Abstract

**Importance:**

Transparency of industry payments to physicians could engender greater public trust in physicians but might also lead to greater mistrust of physicians and the medical profession, adversely affecting the patient-physician relationship.

**Objective:**

To examine the association between nationwide public disclosure of industry payments and Americans’ trust in their physicians and trust in the medical profession.

**Design, Setting, and Participants:**

Survey study using difference-in-difference analyses of a national longitudinal survey comparing changes in states where industry payments were newly disclosed by Open Payments with changes in states where payments information was already available because of state sunshine laws. The US population-based surveys were conducted in September 2014—shortly before the initial public disclosure of industry payments—and again in September 2016. Final analyses were conducted September through December 2018. Participants were adults 18 years and older (n = 1388).

**Exposures:**

National public disclosure through Open Payments of payments made by pharmaceutical and medical device firms to physicians.

**Main Outcomes and Measures:**

Wake Forest measure of trust in one’s own physician and Wake Forest measure of trust in the medical profession.

**Results:**

Of the 3542 original survey respondents, 2180 (61.5%) completed the second survey 2 years later, and 1388 named the same most frequently seen physician in both surveys. The mean age of respondents at the time of the first survey was 53 years, and 749 (54.0%) were women. Race/ethnicity was white in 76.6% (1063 of 1388) and non-Hispanic black in 8.0% (111 of 1388). Public disclosure of payments was associated with lower trust in one’s own physician regardless of whether respondents knew their physicians had received payments (decrease in Wake Forest measure of trust in one’s own physician of 0.56 point; 95% CI, −0.79 to −0.32 point; *P* < .001). Open Payments was also associated with lower trust in the medical profession (decrease in Wake Forest measure of trust in the medical profession of 0.35 point; 95% CI, −0.58 to −0.12 point; *P* = .004).

**Conclusions and Relevance:**

Nationwide public disclosure of industry payments may be associated with decreased trust in physicians and in the medical profession. More judicious presentation of payments information may counteract unintended negative trust and spillover consequences of public disclosure.

## Key Points

**Question:**

Is there an association between nationwide public disclosure of industry payments and Americans’ trust in their physicians?

**Findings:**

In a survey study using a difference-in-difference analysis of a national longitudinal survey of 1388 US adults, public disclosure of payments was associated with respondents reporting lower trust in their own physicians—regardless of whether they knew their physicians had received industry payments—and lower trust in the medical profession.

**Meaning:**

An unintended consequence of the public disclosure of industry payments via Open Payments may have been diminished trust among American individuals in their physicians even though these physicians may not have received any industry payments.

## Teaser

This survey study uses difference-in-difference analysis of national longitudinal survey data to examine the association between nationwide public disclosure of industry payments and Americans’ trust in their physicians and in the medical profession.

## Introduction

Pharmaceutical companies have long cultivated ties with physicians, engaging them through drug detailing, consulting, and research arrangements.^1,2,3^ However, the nature of these relationships and the magnitude of the dollars flowing from companies to physicians have largely been opaque to the public. Concerns about the influence of these financial relationships on physicians’ clinical decisions motivated Congress to include the Physician Payments Sunshine Act (PPSA) in the 2010 Patient Protection and Affordable Care Act.^4,5^ The PPSA requires pharmaceutical and medical device firms to report, for public release, the payments and gifts of monetary value that they make to physicians. This public disclosure requirement was motivated by the belief that patients would become better informed about the potential influence of industry relationships on their physicians’ medical advice. Moreover, transparency might act as a deterrent to physicians accepting potentially suspect payments.^4,6^

However, early studies of patients’ use of the information made available by the PPSA’s Open Payments program have been disappointing. Less than 5% of US adults report knowing about their physicians’ industry payments or using the Open Payments website.^7,8^ These findings are consistent with evaluations of other transparency initiatives in medicine (eg, the public reporting of hospital and physician quality), showing that patients are largely unaware of and rarely use the information disclosed.^9^

Despite low patient engagement rates, public disclosure of industry payments could still have far-reaching consequences for patient well-being by enhancing patient trust in physicians. Open and transparent institutions are believed to engender greater public trust,^10,11^ and supporters of the PPSA consider it an important step toward strengthening trust in the medical profession, which may have been weakened by physicians’ financial relationships with industry.^12,13,14^ Patient trust is thought to influence a wide range of behaviors affecting health, including whether patients follow treatment recommendations, how well they self-manage chronic conditions, and whether they seek preventive care.^15,16,17,18^

However, it is also possible that Open Payments has decreased public trust in physicians. Much of the media coverage of Open Payments has focused on the large amount of industry money directed toward physicians overall and on very large payments received by a minority of physicians.^19,20,21,22^ The critical tone of news media investigations using Open Payments data may have generated a sense of alarm rather than reassurance among patients.

Findings from survey and experimental studies on the relationship between patient trust and disclosure of industry ties have been mixed. Some studies^23,24^ found that patients report higher levels of trust in physicians who disclose their industry relationships, while other studies^25,26,27,28^ demonstrated lower levels of patient trust when physician financial conflicts of interest are disclosed.

For the first time to date, to our knowledge, we sought to explore these trust relationships in a real-world setting. Previous research has focused on the trust consequences of individual-level exposures to information about physician-industry relationships. Efforts to scale up patient exposure to industry payments information have concentrated on making these data available to the public through the Open Payments website.^29^ In this policy setting, some patients may access payments information through the website, some may never be exposed to payments information at all (either by choice or because they are unaware of Open Payments), and others may obtain information in repackaged form through intermediaries like the news media. The overall impact of large-scale transparency initiatives like Open Payments may thus not be readily apparent in smaller-scale studies of individual exposure to payments information.

Through a national longitudinal survey fielded on a cohort of US adults before and after the PPSA’s data release, we investigated changes in respondents’ trust in their physicians and in the medical profession associated with large-scale Open Payments disclosures. Because 3 states (Massachusetts, Minnesota, and Vermont) made industry payments information publicly available before PPSA implementation, respondents in those states served as a comparison group.

## Methods

### Human Participants and Survey Reporting Guidelines

The Drexel University Institutional Review Board determined that the foregoing survey protocol was not research involving human participants as defined by the US Department of Health and Human Services and US Food and Drug Administration guidelines. Reporting of survey elements herein conforms with American Association for Public Opinion Research (AAPOR) survey disclosure guidelines. Respondents indicated their consent by clicking on a screen describing the study and affirming their consent to participate.

### Sample

The sample for the first wave of the longitudinal survey consisted of 3542 American adults 18 years and older selected from KnowledgePanel (KP), a large nationally representative household survey maintained by the research firm GfK. The KP households are selected through address-based sampling so that 97% of US households are covered, including households without internet access. Details on survey sampling methods are provided in eAppendix 1 in the Supplement.

We drew a nationally representative sample, with oversampling in Massachusetts and Minnesota to enable us to detect smaller changes in these 2 states, which had previously passed sunshine laws requiring the public disclosure of industry payments made to physicians in the state. (We did not oversample Vermont, which also had a preexisting disclosure law, because an oversampling of this small population would still not have generated sufficient power to detect a meaningful change in outcome measures in that state.) We refer to these 2 states and Vermont as sunshine states.

The sample for the follow-up survey consisted of the group of all wave 1 respondents who were available for recontact (76.5% [2711 of 3542]). The eFigure in the Supplement shows the flow diagram for sample selection. Individuals who were not available for recontact were more likely than those who were recontacted to be racial/ethnic minorities and to not be in paid employment but were similar along most other dimensions (eTable 1 in the Supplement).

Of the 2711 respondents from wave 1 who were recontacted, 80.4% (2180 of 2711) completed the survey, for an overall completion rate of 61.5% (2180 of 3542). Respondents in the 2 waves were similar on most sociodemographic and health dimensions, and attrition rates did not differ between sunshine and nonsunshine states (eAppendix 1 and eTable 1 in the Supplement). For our analyses, we focused on the subsample of individuals who did not change their physician between the 2 survey waves (n = 1388).

### Survey Design

GfK administered wave 1 and wave 2 surveys online (details are available in eAppendix 1 in the Supplement). Survey questions are provided in eAppendix 2 in the Supplement. Both surveys asked respondents to name the physician they had seen most frequently in the past 12 months.

The 5-item validated Wake Forest measure of trust in one’s own physician^30^ consists of 5 statements related to different dimensions of trust (eg, “I completely trust my doctor’s decisions about which medical treatments are best for me” and “My doctor is extremely thorough and careful”). Respondents rate their agreement or disagreement with each statement on a 5-point scale, and the Wake Forest summary trust score is calculated by summing the 5 responses. Scores thus range from 5 (lowest level of trust) to 25 (highest level of trust). The 5-item validated Wake Forest measure of trust in the medical profession^30,31^ also consists of 5 statements (eg, “A doctor would never mislead me about anything” and “Doctors are extremely thorough and careful”). It is constructed similarly to the Wake Forest measure of trust in one’s own physician, with summary score values ranging from 5 to 25.

Using 5-point Likert-type scales, respondents were also asked to rate their agreement with the statement “My doctor is a real expert in taking care of medical problems like mine” and their satisfaction with the care they received from their named physician. They were also asked if they knew whether their physician had received any industry payments. Survey data were linked to information on respondents’ sociodemographic and self-reported health characteristics provided by GfK.

### Survey Administration

The US population-based surveys were conducted in September 2014—shortly before the initial public disclosure of industry payments—and again in September 2016. The first survey was fielded September 26 to October 3, 2014, with almost all surveys (90.5% [3206 of 3542]) completed by the Open Payments data release date of September 30. The wave 2 survey was fielded September 16 to October 2, 2016, approximately 2 years after the initial survey.

### Outcome Measures

There were 4 outcomes of interest. These were (1) the Wake Forest summary score for trust in one’s own physician, (2) the Wake Forest summary score for trust in the medical profession, (3) the rating of the expertise of one’s own physician, and (4) the rating of satisfaction with the care provided by one’s own physician.

### Statistical Analysis

We used a difference-in-difference approach (interrupted time series with comparison group)^32^ to estimate the association between the national public release of Open Payments information and the outcome measures. In the sample of respondents who did not change physicians between wave 1 and wave 2, we compared changes in the outcome measures between 2014 and 2016 among residents of states without sunshine laws with changes during the same period among residents of sunshine states (Massachusetts, Minnesota, and Vermont), which had previously disclosed payments information. The use of sunshine states as comparisons improves on a simple pre-post design and accounts for secular trends influencing all states that otherwise would have confounded our estimates.

We estimated difference-in-difference associations in 3 samples. We first estimated a model using the full sample of 50 states to obtain an overall national estimate. However, because the 3 sunshine states may not be ideal comparisons for all of the remaining 47 states, we then estimated associations in more geographically comparable areas. Specifically, we estimated associations in states in the same census region as Minnesota using Minnesota as the comparison state; states in the Midwest region include Illinois, Indiana, Iowa, Kansas, Michigan, Missouri, Nebraska, North Dakota, Ohio, South Dakota, and Wisconsin. We also estimated associations in states in the same census region as Massachusetts and Vermont using these 2 states as the comparison states. States in the Northeast region include Connecticut, Maine, New Hampshire, New Jersey, New York, Pennsylvania, and Rhode Island.

We calculated unadjusted and adjusted difference-in-difference estimates. Regression-adjusted models—used to increase precision of the estimates—included sex, age, education, race/ethnicity, household income, employment status, urban or rural residence, previous diagnoses (separate indicators for any chronic condition, mental health disorder, cancer, and stroke or myocardial infarction), whether insured, number of physician visits, quadratic terms of age and number of physician visits to account for nonlinearities, and knowledge of whether one’s own physician had received industry payments (an indicator of knowledge that one’s physician had received payments and an indicator of knowledge that one’s own physician had not received payments, with the reference category of no knowledge of whether one’s own physician had received payments). Standard errors were clustered at the state level.

We computed 95% CIs for all estimates and conducted 2-tailed *t* tests of statistical significance, where *P* < .05 was considered indicative of statistical significance. Descriptive and regression analyses were conducted September through December 2018 using Stata version 14 (StataCorp LP).

## Results

### Sample Characteristics

Of the 3542 original survey respondents, 2180 (61.5%) completed the second survey 2 years later, and 1388 named the same most frequently seen physician in both surveys. In the group of respondents who did not change physicians, the mean age at the time of the first survey was 53 years. In total, 749 of 1388 (54.0%) were women, 76.6% (1063 of 1388) were white, and 8.0% (111 of 1388) were non-Hispanic black.

Approximately 90% (1244 of 1388) of respondents lived in nonsunshine states (ie, were newly exposed to industry payments information), and 10.4% (144 of 1388) lived in sunshine states (Table 1). Respondents in the 2 types of states were similar with regard to self-reported health conditions and most demographic characteristics, although residents of sunshine states were more likely to be of white race/ethnicity and employed and had higher household income.

**Table 1.** Characteristics of 1388 Respondents at Baseline, 2014

Characteristic	No. (%)		*P* Value^c^
	Nonsunshine State^a^	Sunshine State^a^^,^^b^	
No. of respondents	1244 (100)	144 (100)	NA
Sex			
Male	566 (45.5)	73 (50.7)	.24
Female	678 (54.5)	71 (49.3)	
Age, y			
≤20	25 (2.0)	0	.26
21-30	137 (11.0)	11 (7.6)	
31-40	167 (13.4)	17 (11.8)	
41-50	171 (13.7)	18 (12.5)	
51-60	302 (24.3)	43 (29.9)	
≥61	442 (35.5)	55 (38.2)	
Education			
Less than high school	96 (7.7)	5 (3.5)	.05
High school graduate	410 (33.0)	42 (29.2)	
Some college	360 (28.9)	39 (27.1)	
College graduate	378 (30.4)	58 (40.3)	
Race/ethnicity			
White	933 (75.0)	130 (90.3)	<.001
Hispanic	116 (9.3)	4 (2.8)	
Black, non-Hispanic	108 (8.7)	3 (2.1)	
Other	87 (7.0)	7 (4.9)	
Household income, $			
0-24 999	210 (16.9)	18 (12.5)	.04
25 000-49 999	283 (22.7)	22 (15.3)	
50 000-74 999	247 (19.9)	28 (19.4)	
75 000-99 999	162 (13.0)	21 (14.6)	
≥100 000	342 (27.5)	55 (38.2)	
Employment status			
Employed for pay	585 (47.0)	76 (52.8)	.03
Self-employed	73 (5.9)	11 (7.6)	
Retired	337 (27.1)	44 (30.6)	
Not working–disability	93 (7.5)	6 (4.2)	
Not working–other	156 (12.5)	7 (4.9)	
Urban or rural residence			.25
Urban	192 (15.4)	17 (11.8)	
Rural	1052 (84.6)	127 (88.2)	
Self-rated health^d^			.38
Excellent	150 (12.1)	22 (15.3)	
Good	790 (63.5)	96 (66.7)	
Fair	262 (21.1)	23 (16.0)	
Poor	34 (2.7)	3 (2.1)	
Diagnosis of any chronic condition^e^^,^^f^			
No	432 (34.7)	47 (32.6)	.64
Yes	808 (65.0)	96 (66.7)	
Diagnosis of mental health disorder^f^			
No	1032 (83.0)	124 (86.1)	.29
Yes	208 (16.7)	19 (13.2)	
Diagnosis of cancer^f^			
No	1100 (88.4)	129 (89.6)	.59
Yes	140 (11.3)	14 (9.7)	
Diagnosis of stroke or myocardial infarction^f^			
No	1186 (95.3)	138 (95.8)	.63
Yes	54 (4.3)	5 (3.5)	
Any health insurance coverage^g^			
No	160 (12.9)	12 (8.3)	.12
Yes	1084 (87.1)	132 (91.7)	

Abbreviation: NA, not applicable.

^a^ Nonsunshine refers to states that had not publicly disclosed industry payments information before Open Payments. Sunshine refers to states that had (because of state statute) disclosed industry payments information before Open Payments.

^b^ Includes 79 respondents in Massachusetts and Vermont and 65 respondents in Minnesota.

^c^ *P* value from χ^2^ test of independence comparing distributions in nonsunshine states with distributions in sunshine states.

^d^ Includes 1236 respondents in nonsunshine states and 144 respondents in sunshine states.

^e^ Chronic conditions include acid reflux, asthma, atrial fibrillation, chronic obstructive pulmonary disease, chronic pain, cystic fibrosis, diabetes, epilepsy, eye disease, gout, heart disease, hepatitis C, hypertension, high cholesterol, HIV, kidney disease, multiple sclerosis, osteoarthritis, osteoporosis, rheumatoid arthritis, and sleep disorder.

^f^ Includes 1240 respondents in nonsunshine states and 143 respondents in sunshine states.

^g^ Includes 1244 respondents in nonsunshine states and 144 respondents in sunshine states.

### Disclosure and Trust in One’s Own Physician

Table 2 summarizes the mean respondent levels of trust in one’s own physician before disclosure, as well as the unadjusted and adjusted difference-in-difference associations for the full national sample, the Northeast sample, and the Midwest sample. There was little difference between the unadjusted and adjusted estimates of the association between public disclosure and trust, with adjusted estimates more precisely estimated.

**Table 2.** Changes in Wake Forest Measure of Trust in One’s Own Physician After Payments Information Disclosure^a^

Region	Wake Forest Trust Scale Predisclosure Mean (SE)	Unadjusted Estimate		Adjusted Estimate^b^	
		Change (95% CI)	*P* Value	Change (95% CI)	*P* Value
United States (vs Massachusetts, Minnesota, and Vermont)					
Nonsunshine (n = 1244)^c^	20.5 (0.1)	−0.58 (−0.77 to −0.40)	<.001	−0.56 (−0.79 to −0.32)	<.001
Sunshine comparison (n = 144)^c^^,^^d^	20.5 (0.3)				
Northeast (vs Massachusetts and Vermont)					
Nonsunshine (n = 220)^c^	20.8 (0.2)	−0.58 (−1.20 to 0.03)	.06	−0.44 (−0.91 to 0.03)	.06
Sunshine comparison (n = 79)^c^^,^^e^	20.5 (0.4)				
Midwest (vs Minnesota)					
Nonsunshine (n = 298)^c^	20.7 (0.2)	−0.67 (−0.95 to −0.39)	<.001	−0.61 (−1.01 to −0.22)	.006
Sunshine comparison (n = 65)^c^^,^^f^	20.6 (0.4)				

^a^ All analyses are based on a balanced panel of individuals who did not change physicians between wave 1 and wave 2. On the Wake Forest Measure of Trust in One’s Own Physician, 5 indicates lowest trust, and 25 indicates highest trust.

^b^ Adjusted models include sex, age, education, race/ethnicity, household income, employment status, urban or rural residence, previous diagnosis of any chronic condition (including acid reflux, asthma, atrial fibrillation, chronic obstructive pulmonary disease, chronic pain, cystic fibrosis, diabetes, epilepsy, eye disease, gout, heart disease, hepatitis C, hypertension, high cholesterol, HIV, kidney disease, multiple sclerosis, osteoarthritis, osteoporosis, rheumatoid arthritis, and sleep disorder), previous diagnosis of mental health disorder, previous diagnosis of cancer, previous diagnosis of stroke or myocardial infarction, whether insured, number of visits to the physician, quadratic terms of age and number of visits to account for nonlinearities in age and number of visits, and knowledge of whether one’s own physician had received industry payments. Standard errors were clustered at the state level.

^c^ Nonsunshine refers to states that had not publicly disclosed industry payments information before Open Payments. Sunshine refers to states that had (because of state statute) disclosed industry payments information before Open Payments.

^d^ Sunshine comparison states for the United States include Massachusetts, Minnesota, and Vermont.

^e^ Sunshine comparison states for the Northeast include Massachusetts and Vermont.

^f^ Sunshine comparison state for the Midwest includes Minnesota.

In the 50-state adjusted comparison, Open Payments was associated with a statistically significant 0.56-point decline in the summary trust measure (b = −0.56; 95% CI, −0.79 to −0.32; *P* < .001) (Table 2). The difference was also significant for the Midwest states comparison (b = −0.61; 95% CI, −1.01 to −0.22; *P* = .006) but did not reach the .05 significance level for the Northeast states comparison (b = −0.44; 95% CI, −0.91 to 0.03; *P* = .06).

### Disclosure and Trust in the Medical Profession

Respondents reported greater mean levels of trust in their own physician than in the medical profession in general, with scores of 20.5 (95% CI, 20.3-20.7) vs 15.4 (95% CI, 15.2-15.5) on the Wake Forest Trust Scale (approximately a 5-point mean difference). Table 3 lists the mean levels of trust in the medical profession before disclosure, as well as the unadjusted and adjusted difference-in-difference estimates of the association between Open Payments and trust in the medical profession.

**Table 3.** Changes in Wake Forest Measure of Trust in the Medical Profession After Payments Information Disclosure^a^

Region	Wake Forest Trust Scale Predisclosure Mean (SE)	Unadjusted Estimate		Adjusted Estimate^b^	
		Change (95% CI)	*P* Value	Change (95% CI)	*P* Value
United States (vs Massachusetts, Minnesota, and Vermont)					
Nonsunshine (n = 1244)^c^	15.3 (0.1)	−0.23 (−0.42 to −0.05)	.02	−0.35 (−0.58 to −0.12)	.004
Sunshine comparison (n = 144)^c^^,^^d^	16.2 (0.3)				
Northeast (vs Massachusetts and Vermont)					
Nonsunshine (n = 220)^c^	15.5 (0.2)	−0.30 (−0.84 to 0.24)	.23	−0.34 (−0.96 to 0.27)	.23
Sunshine comparison (n = 79)^c^^,^^e^	15.9 (0.4)				
Midwest (vs Minnesota)					
Nonsunshine (n = 298)^c^	15.4 (0.2)	−0.24 (−0.62 to 0.13)	.18	−0.42 (−1.02 to 0.17)	.15
Sunshine comparison (n = 65)^c^^,^^f^	16.4 (0.5)				

^a^ All analyses are based on a balanced panel of individuals who did not change physicians between wave 1 and wave 2. On the Wake Forest Measure of Trust in the Medical Profession, 5 indicates lowest trust, and 25 indicates highest trust.

^b^ Adjusted models include sex, age, education, race/ethnicity, household income, employment status, urban or rural residence, previous diagnosis of any chronic condition (which include acid reflux, asthma, atrial fibrillation, chronic obstructive pulmonary disease, chronic pain, cystic fibrosis, diabetes, epilepsy, eye disease, gout, heart disease, hepatitis C, hypertension, high cholesterol, HIV, kidney disease, multiple sclerosis, osteoarthritis, osteoporosis, rheumatoid arthritis, and sleep disorder), previous diagnosis of mental health disorder, previous diagnosis of cancer, previous diagnosis of stroke or myocardial infarction, whether insured, number of visits to the physician, quadratic terms of age and number of visits to account for nonlinearities in age and number of visits, and knowledge of whether one’s own physician had received industry payments. Standard errors were clustered at the state level.

^c^ Nonsunshine refers to states that had not publicly disclosed industry payments information before Open Payments. Sunshine refers to states that had (because of state statute) disclosed industry payments information before Open Payments.

^d^ Sunshine comparison states for the United States include Massachusetts, Minnesota, and Vermont.

^e^ Sunshine comparison states for the Northeast include Massachusetts and Vermont.

^f^ Sunshine comparison state for the Midwest includes Minnesota.

Open Payments was associated with a statistically significant 0.35-point decline in the mean levels of trust in the medical profession in the 50-state adjusted comparison (b = −0.35; 95% CI, −0.58 to −0.12; *P* = .004) (Table 3). There were also declines in trust in the medical profession in the 2 regional comparisons, but these did not reach statistical significance.

### Disclosure and Rating of Physician’s Expertise and Satisfaction With Care

Table 4 summarizes the unadjusted and adjusted difference-in-difference results related to respondents’ ratings of physician expertise and respondents’ satisfaction with the health care received from their physician. Nationally and regionally, there were declines in ratings of physician expertise and care satisfaction, but the differences between sunshine and nonsunshine states were statistically significant only in the Midwest comparison. In that region, there was an 11.9–percentage point decline in the percentage of respondents who rated their physician’s expertise highly (b = −11.9%; 95% CI, −17.8% to −6.0%; *P* = .001) and a 6.7–percentage point decline in the percentage of respondents who were satisfied or very satisfied with the care their physician provided (b = −6.7%; 95% CI, −13.0% to −0.4%; *P* = .04).

**Table 4.** Changes in Ratings of Physician Expertise and Satisfaction With Health Care After Payments Information Disclosure^a^

Region	Predisclosure % (SE)	Unadjusted Estimate		Adjusted Estimate^b^	
		Percentage Point Change (95% CI)	*P* Value	Percentage Point Change (95% CI)	*P* Value
**Physician Expertise (% Agree or Strongly Agree: My Doctor Is a Real Expert in Taking Care of Medical Problems Like Mine)**					
United States (vs Massachusetts, Minnesota, and Vermont)					
Nonsunshine (n = 1244)^c^	79 (1)	−4.7 (−13.2 to 3.8)	.27	−4.9 (−15.2 to 5.4)	.34
Sunshine comparison (n = 144)^c^^,^^d^	79 (3)				
Northeast (vs Massachusetts and Vermont)					
Nonsunshine (n = 220)^c^	82 (3)	−2.0 (−5.5 to 1.6)	.23	−1.4 (−4.4 to 1.6)	.31
Sunshine comparison (n = 79)^c^^,^^e^	82 (4)				
Midwest (vs Minnesota)					
Nonsunshine (n = 298)^c^	79 (2)	−11.4 (−16.1 to −6.7)	<.001	−11.9 (−17.8 to −6.0)	.001
Sunshine comparison (n = 65)^c^^,^^f^	76 (5)				
**Satisfaction With Health Care (% Satisfied or Very Satisfied: In General, How Satisfied Are You With the Health Care You Received in the Past 12 Months From Your Doctor?)**					
United States (vs Massachusetts, Minnesota, and Vermont)					
Nonsunshine (n = 1244)^c^	83 (1)	−2.9 (−5.8 to −0.1)	.04	−3.0 (−6.4 to 0.4)	.08
Sunshine comparison (n = 144)^c^^,^^d^	87 (3)				
Northeast (vs Massachusetts and Vermont)					
Nonsunshine (n = 220)^c^	83 (2)	−2.3 (−9.4 to 4.9)	.48	−2.9 (−8.9 to 3.1)	.30
Sunshine comparison (n = 79)^c^^,^^e^	90 (3)				
Midwest (vs Minnesota)					
Nonsunshine (n = 298)^c^	86 (2)	−5.8 (−11.4 to −0.1)	.05	−6.7 (−13.0 to −0.4)	.04
Sunshine comparison (n = 65)^c^^,^^f^	83 (5)				

^a^ All analyses are based on a balanced panel of individuals who did not change physicians between wave 1 and wave 2.

^b^ Adjusted models include sex, age, education, race/ethnicity, household income, employment status, urban or rural residence, previous diagnosis of any chronic condition (which include acid reflux, asthma, atrial fibrillation, chronic obstructive pulmonary disease, chronic pain, cystic fibrosis, diabetes, epilepsy, eye disease, gout, heart disease, hepatitis C, hypertension, high cholesterol, HIV, kidney disease, multiple sclerosis, osteoarthritis, osteoporosis, rheumatoid arthritis, and sleep disorder), previous diagnosis of mental health disorder, previous diagnosis of cancer, previous diagnosis of stroke or myocardial infarction, whether insured, number of visits to the physician, quadratic terms of age and number of visits to account for nonlinearities in age and number of visits, and knowledge of whether one’s own physician had received industry payments. Standard errors were clustered at the state level.

^c^ Nonsunshine refers to states that had not publicly disclosed industry payments information before Open Payments. Sunshine refers to states that had (because of state statute) disclosed industry payments information before Open Payments.

^d^ Sunshine comparison states for the United States include Massachusetts, Minnesota, and Vermont.

^e^ Sunshine comparison states for the Northeast include Massachusetts and Vermont.

^f^ Sunshine comparison state for the Midwest includes Minnesota.

### Trust and Knowledge of Whether One’s Own Physician Received Payments

In all of the adjusted regression models, we included an indicator of knowledge that one’s own physician had received industry payments and an indicator of knowledge that one’s own physician had not received industry payments (the reference category was the respondent not knowing if his or her physician had received payments). Because approximately only 3% of respondents reported knowing whether their physicians had received any industry payments, the coefficients on these variables are determined by a small group of respondents. In the national sample, knowing that one’s own physician had not received industry payments was associated with 1.56 points greater mean levels of trust in one’s own physician (b = 1.56; 95% CI, 0.56-2.56; *P* = .003) relative to not knowing whether one’s own physician had received any payments (eTable 2 in the Supplement).

## Discussion

Although there was reason to believe that increasing transparency about physicians’ financial relationships with industry could enhance public trust in medicine and in physicians, we found that patients’ trust in their own physicians and in the medical profession decreased after the release of Open Payments data. The decline in the Wake Forest measure of trust in one’s own physician—0.56 points from an initial mean level of 20.5—corresponds to a 2.7% decline in trust.

Although modest in absolute terms, this decrease is comparable in magnitude to the trust associations reported in studies of the disclosure of managed care incentives. In the 1990s and early 2000s, the emergence of managed care payment models and their focus on reducing health care costs, possibly at the expense of quality, raised concerns about conflicts of interest and loss of trust in physicians. Studies^33,34^ reported declines in trust of 0% to 4% attributable to knowing about managed care payment regimens.

Consistent with previous research,^31,34^ we found that Americans’ trust in the medical profession was substantially lower than their trust in their own physician. Individuals draw a clear distinction between their expectations of their own physician—whom they have personally selected among a range of choices—and their expectations of physicians in general. This may explain the smaller declines in trust in the profession as a whole, statistically detectable only in the larger 50-state sample.

Significantly, respondents appear to be reporting less trust in their own physician regardless of whether the physician had industry ties. Because few respondents knew whether their physician had received industry payments and about 65% of respondents visited physicians who received payments,^35^ respondents reported diminished trust even though their physicians may not have received any payments. Therefore, public disclosure may have resulted in negative reputational spillovers affecting “pharma-free” physicians^36^ and physicians with industry ties.

What might explain the observed decline in trust, particularly given respondents’ low awareness of their own physician’s payments record? One possibility is the media reporting of Open Payments, which has characterized industry payments as pervasive and large and focused on the minority of physicians who have received very large payments.^19,20,21,22^ The attention paid to extreme cases may have led to patients consciously or unconsciously shifting their perceptions of their own physician and of physicians in general.

We observed little association between Open Payments and respondents’ ratings of their physician’s expertise or in the satisfaction with their care, except among states in the Midwest census region. Residents of Midwest states newly exposed to payments information also showed greater declines in trust in their physicians than their counterparts in the Northeast. One reason for this geographic heterogeneity may be that in the Northeast—which consists of states with small land areas and densely populated regions crossing state lines—diffusion of information regarding industry payments to physicians may have already been widespread at the time of the public release of the national data. For example, coverage of the Massachusetts and Vermont sunshine laws in *The Boston Globe* and on boston.com, which are read throughout the New England region, may have contributed to spillover effects across states. Therefore, the release of Open Payments and the reporting around the federal program may not have provided much additional information in this region.

### Policy Implications

Trust is a crucial element of the physician-patient relationship affecting many aspects of patient behavior and sentiment that ultimately affect health. Patient trust in their physicians has been shown to be associated with adherence to treatment, disease self-management, and care satisfaction and is thought to be associated with care-seeking behavior and the use of preventive care services.^15,16,17,18^ Trust in the medical profession may affect the public’s views of scientific authority and medical research, which may influence patient adherence and health-promoting behaviors. Our finding that the nationwide disclosure of industry payments was associated with reduced trust in physicians portends negative consequences of disclosure for the public’s health.

These negative trust consequences of public disclosure need not imply that, as a policy matter, patients should not be informed of industry payments. Indeed, diminished trust could be warranted in some cases. Some physicians may be less trustworthy because of their financial ties to industry, and journalists and researchers should investigate these financial relationships.

However, our findings raise the troubling prospect of knowledge that some physicians accept industry payments may lead patients to paint their physicians with a broad brush, mistrusting them generally rather than in response to actual instances of financial ties. Some research has shown that patients report less trust in a physician when they know the physician has industry ties.^25,26,27,28^ Our findings suggest a more worrying prospect of diminished trust in physicians who do not receive industry payments because patients are aware that some physicians do.

To minimize such spillover consequences, institutional policies could help patients identify which physicians have industry ties and—because not all industry ties are equally concerning—explain the specific nature of these ties to provide context. In addition, physicians with no industry ties could take the initiative to advertise that status to current and prospective patients. Policymakers, journalists, and scholars should also take care to set their discussions of physicians who receive very high payments in the context of the broader population of physicians. Patients should understand that such cases are not the norm: the median annual payment to physicians was $201 in the 2015 Open Payments data.^37^

### Limitations

This study has several limitations. Our statistical strategy allows for stronger inferences to be drawn about the association between payments disclosure and trust than a simple pre-post design because it uses a comparison group. However, we cannot rule out confounding if there were other changes in the health care environment affecting trust, expertise, or care satisfaction that differentially affected sunshine and nonsunshine states between 2014 and 2016. Furthermore, in restricting the sample to individuals who responded to both predisclosure and postdisclosure surveys (ie, who were not lost to attrition) and who did not change their most frequently seen physician between the 2 waves, our sample is not fully representative of US households. Also, the regional comparisons are based on a relatively small number of survey respondents in sunshine states.

## Conclusions

Transparency as a means to support informed patient decision making appears to be a sound principle, but the potential reputational spillover effects from noisy information may prevent transparency from achieving its goal of fully informing patients. Going forward, it is important to focus research and policy efforts not simply on more transparency but on more trust building and more effective transparency. For example, are effects on trust different when the same information is disclosed by the physician personally vs when the patient learns of the physician’s industry payments through other channels? How, if at all, do effects differ when the physician’s disclosure is voluntary vs mandatory? Further opportunities abound to strengthen emerging practices of transparency in this important area.

## Notes

Supplement. **eAppendix 1.** Survey Sampling Methods **eAppendix 2.** Survey Questions **eFigure.** Sample Selection Flow Diagram **eTable 1.** Characteristics of Wave 1 Respondents, Wave 2 Respondents, and Non-Respondents **eTable 2.** Coefficients Associated With Knowledge of Whether Own Physician Received Payments
