# Supplementary material for: US Nationwide Disclosure of Industry Payments and Public Trust in Physicians
Source: JAMA Netw Open. 2019 Apr 12;2(4):e191947. doi: 10.1001/jamanetworkopen.2019.1947 (PMC6481437; doi:10.1001/jamanetworkopen.2019.1947)
Supplement: Supplement. — eAppendix 1. Survey Sampling Methods eAppendix 2. Survey Questions eFigure. Sample Selection Flow Diagram eTable 1. Characteristics of Wave 1 Respondents, Wave 2 Respondents, and Non-Respondents eTable 2. Coefficients Associated With Knowledge of Whether Own Physician Received Payments [file jamanetwopen-2-e191947-s001.pdf]

Supplementary Online Content

Kanter GP, Carpenter D, Lehmann LS, Mello MM. US nationwide disclosure of industry payments and public trust in physicians. *JAMA Netw Open*. 2019;2(4):e191947. doi:10.1001/jamanetworkopen.2019.1947

eAppendix 1. Survey Sampling Methods

eAppendix 2. Survey Questions

eFigure. Sample Selection Flow Diagram

eTable 1. Characteristics of Wave 1 Respondents, Wave 2 Respondents, and Non-Respondents

eTable 2. Coefficients Associated With Knowledge of Whether Own Physician Received Payments

This supplementary material has been provided by the authors to give readers additional information about their work.

© 2019 Kanter GP et al. *JAMA Network Open*.

eAppendix 1. Survey Sampling Methods

Survey Sampling and Administration

Individuals selected for the initial (Wave 1) survey were recruited from KnowledgePanel® (KP), a nationally representative household panel assembled by the research firm GfK. KP households are selected through random digit dialing and address-based sampling so that landline households as well as cell-phone-only and no-phone households are in a sampling frame covering 97% of US households. KP households agree to participate in occasional surveys and, in return for their participation, accumulate points that they can redeem for cash, merchandise, and other items of monetary value (average accumulation valued at $4-$6 per month). Households without Internet access are provided a web-enabled computer and free Internet service for the duration of their participation in the panel. Detailed information about KP sampling methodology, incentive structures, informed consent, and other human subjects issues are documented in Dennis and Thomas (2013).[1]

For Wave 1, individuals were sampled from KP households in all 50 states, excluding DC, to constitute a nationally representative sample, with oversampling in Massachusetts and Minnesota to enable us to detect smaller effects in these two states that had previously passed Sunshine laws. We did not oversample Vermont, the third Sunshine state, because even an oversample of this relatively small population would not have generated sufficient power to detect an effect in that state.

The Wave 1 sample consisted of 3,542 respondents who completed the initial survey in 2014 (Wave 1 completion 45.9%). More details on administration of the Wave 1 survey may be found in Pham-Kanter et al (2017).[2]

For Wave 2, GfK identified 2,711 (77%) respondents from Wave 1 respondents who were still in the panel in 2016 and who were available for re-contact. All of these individuals were asked to complete the Wave 2 survey.

Survey Field Period

The first survey was fielded online September 26-October 3, 2014, with almost all surveys (91%) completed by the Open Payments data release date of September 30. The Wave 2 survey was fielded online September 16-October 2, 2016, two years after the initial survey.

Individuals selected for the surveys received a notification email with a link to the survey. After three days, individuals who had not responded to the survey were sent an email reminder. For Wave 2, which had a slightly longer field period than Wave 1, non-respondents also received an automated email reminder 11 days after the initial survey contact.

© 2019 Kanter GP et al. *JAMA Network Open*.

Completion Rate

Of those who were re-contacted for Wave 2, 80% (n=2,180) completed the survey, resulting in an overall completion rate of 62%. The sample selection flow diagram is shown in the eFigure.

Completion rates varied by state and ranged from 40% to 81.8%. Average completion rates for Sunshine and non-Sunshine states did not differ significantly from each other (61.1% and 66.5%, respectively, p=0.06).

Survey Weights

GfK provided survey weights that accounted for non-coverage, oversampling, non-response, and attrition. Although we did not use survey weights for the main analysis—which relied on the oversample of Massachusetts and Minnesota respondents—we used survey weights to compute the distributions of respondent and non-respondent characteristics shown in eTable 1 to make them more easily comparable to known distributions of demographic characteristics of the US population.

GfK weights use, as a benchmark, distributions derived from the 2014 March Supplement Current Population Survey so the survey sample matches the US adult population on key demographic dimensions (gender, age, race/Hispanic ethnicity, education, Census region, household income, homeownership status, metropolitan residence, Internet access). Details on the construction of survey weights are documented in Dennis and Thomas (2013).[1,3]

References

1. Dennis JM, Thomas RK. Documentation for human subjects review committees: GfK company information, past external review, confidentiality, and privacy protections for panelists. Updated 2013.
2. Pham-Kanter G, Mello MM, Lehmann LS, Campbell EG, Carpenter D. Public awareness of and contact with physicians who receive industry payments: a national survey. J Gen Intern Med 2017; 32:767-774.
3. KnowledgePanel® recruitment and sample survey methodologies. https://www.gfk.com/fileadmin/user_upload/dyna_content/US/documents/KnowledgePanel_Methodology.pdf. Undated. Accessed February 17, 2019.

© 2019 Kanter GP et al. *JAMA Network Open*.

eAppendix 2. Survey Questions

Wake Forest Measure of Trust in Own Physician

We would like to ask you questions about the trust and confidence you have in your doctor. Please state whether you strongly agree, agree, neither agree nor disagree, disagree, or strongly disagree with the following statements.

- I completely trust [*name of respondent's doctor* or my doctor]'s decisions about which medical treatments are best for me.
- [*Name of respondent's doctor* or My doctor] is totally honest in telling me about all the different treatment options available for my condition.
- Sometimes [*name of respondent's doctor* or my doctor] cares more about what is convenient for him or her than about my medical needs.
- [*Name of respondent's doctor* or My doctor] is extremely thorough and careful.
- All in all, I have complete trust in [*name of respondent's doctor* or my doctor].

Physician Expertise

- *[Name of respondent's doctor* or My doctor] is a real expert in taking care of medical problems like mine.

Wake Forest Measure of Trust in Medical Profession

We would like to ask you questions about the trust and confidence you have in the medical profession. Please state whether you strongly agree, agree, neither agree nor disagree, disagree, or strongly disagree with the following statements.

- A doctor would never mislead me about anything.
- I complete trust doctors' decisions about which medical treatments are best for me.
- Sometimes doctors care more about what is convenient for him or her than about my medical needs.
- Doctors are extremely thorough and careful.
- All in all, I trust doctors completely.

© 2019 Kanter GP et al. *JAMA Network Open*.

Satisfaction with Health Care

In general, how satisfied are you with the health care you received in the past 12 months from [*name of respondent's doctor* or your doctor]?

Response choices:
Very satisfied
Somewhat satisfied
Somewhat dissatisfied
Very dissatisfied
I haven't had health care from my doctor in the past 12 months

Knowledge of Whether Own Physician Has Received Industry Payments

Do you know whether [*name of respondent's doctor* or your doctor] has received any payments from a pharmaceutical or medical device firm?

Response choices:
Yes, I know my doctor has received payments
Yes, I know my doctor has not received any payments
No, I do not know whether my doctor has received any payments
Not sure

© 2019 Kanter GP et al. *JAMA Network Open*.

eFigure. Sample Selection Flow Diagram

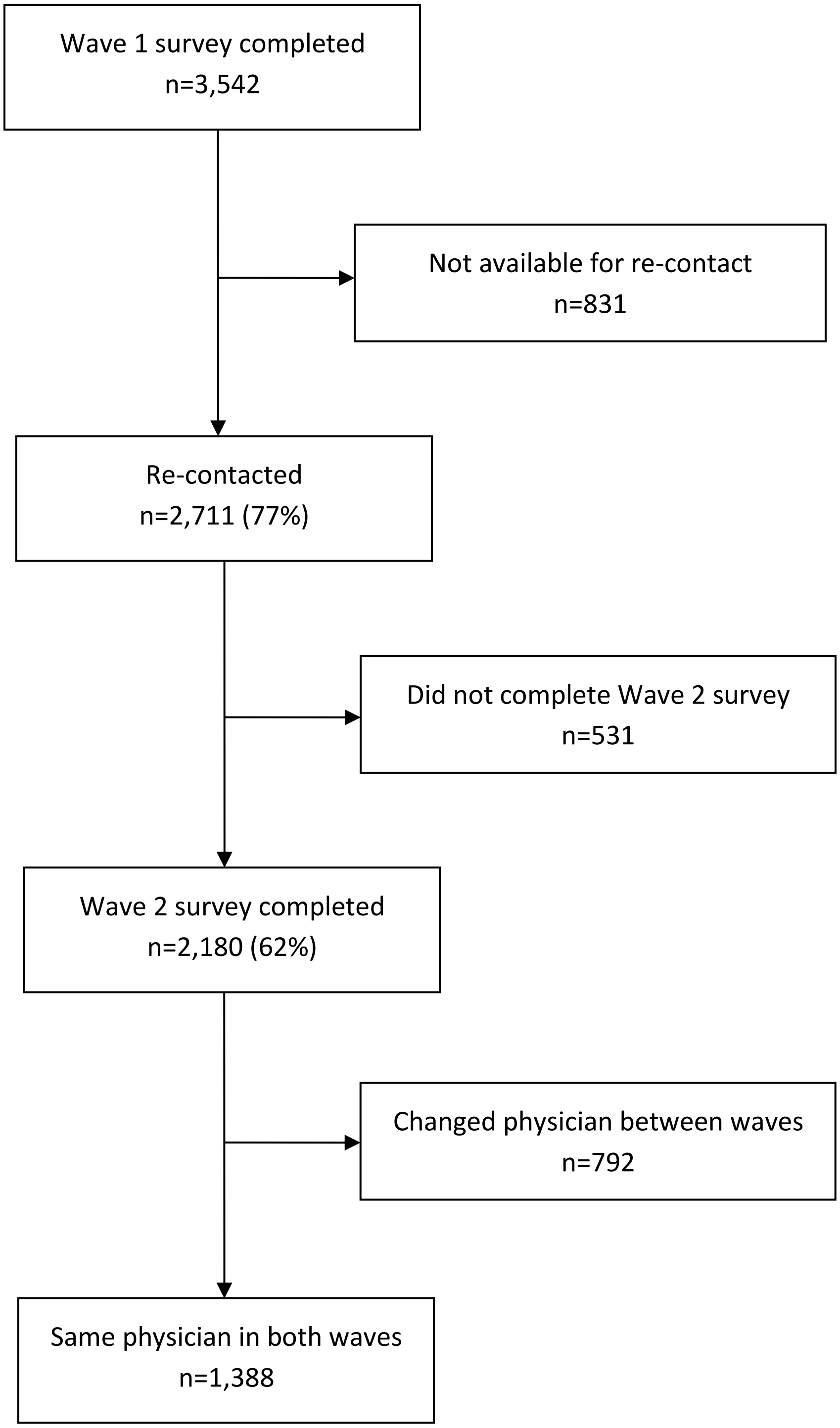

© 2019 Kanter GP et al. *JAMA Network Open*.

eTable 1. Characteristics of Wave 1 Respondents, Wave 2 Respondents, and Non-Respondents

	Weighted Distribution %[b]		
	Wave 1 (2014) n=3,542	Wave 2 (2016) n=2,180	Not contacted or did not respond n=1,362
Gender			
Female	52%	52%	56%
Male	48%	48%	44%
Race/Ethnicity			
Caucasian	66%	65%	60%
Hispanic	15%	15%	14%
Black, Non-Hispanic	11%	12%	18%
Other	8%	8%	8%
Age			
<=20	4%	2%	5%
21-30	19%	18%	23%
31-40	16%	17%	16%
41-50	15%	17%	16%
51-60	21%	21%	18%
61+	25%	25%	22%
Education			
Less than high school	12%	10%	15%
High school graduate	30%	29%	28%
Some college	29%	29%	30%
College graduate	29%	32%	27%
Household Income			
$0 - $24,999	18%	17%	21%
$25,000 - $49,999	23%	21%	24%
$50,000 - $74,999	18%	18%	18%
$75,000 - $99,999	15%	14%	14%
$100,000+	26%	30%	23%
Employment			
Employed for pay	50%	57%	48%
Self-employed	7%	6%	7%
Retired	19%	18%	16%
Not working - disability	7%	6%	8%
Not working - other	17%	13%	21%
Urban/Rural			
Urban	84%	86%	84%
Rural	16%	14%	16%
Resides in state with Sunshine Law			

© 2019 Kanter GP et al. *JAMA Network Open*.

No	96%	96%	96%
Yes	4%	4%	4%
Self-rated Health			
Excellent	14%	13%	15%
Good	61%	64%	58%
Fair	21%	20%	22%
Poor	4%	3%	5%
Diagnosis of chronic condition[c]			
No	45%	46%	49%
Yes	55%	54%	51%
Diagnosis of mental health disorder			
No	82%	98%	80%
Yes	18%	2%	20%
Diagnosis of cancer			
No	91%	94%	92%
Yes	9%	6%	8%
Diagnosis of stroke or myocardial infarction			
No	97%	95%	96%
Yes	3%	5%	4%
Any health insurance coverage			
No	18%	8%	21%
Yes	82%	92%	79%

Notes:

a. Respondent characteristics from demographic profile maintained by GfK.

b. These population-weighted distributions are not directly comparable to the unweighted distributions in Table 1 because the main analysis uses the unweighted oversample of Massachusetts and Minnesota residents.

c. Chronic conditions include acid reflux, asthma, atrial fibrillation, COPD, chronic pain, cystic fibrosis, diabetes, epilepsy, eye disease, gout, heart disease, hepatitis C, hypertension, high cholesterol, HIV, kidney disease, multiple sclerosis, osteoarthritis, osteoporosis, rheumatoid arthritis, sleep disorder.

© 2019 Kanter GP et al. *JAMA Network Open*.

eTable 2. Coefficients Associated With Knowledge of Whether Own Physician Received Payments[a]

	US (vs. MA, MN, & VT) n=1,244 (vs. n=144)		Northeast (vs. MA & VT) n=220 (vs. n=79)		Midwest (vs. MN) n=298 (vs. n=65)	
Independent variable[b]	b (95% CI)	*p* value	b (95% CI)	*p* value	b (95% CI)	*p* value
**Wake Forest measure of trust in own physician (5=lowest trust, 25=greatest trust)**						
Do not know if my doctor received payments	ref		ref		ref	
Know my doctor received payments	-1.23 (-2.8,0.33)	0.12	-0.47 (-1.62,0.68)	0.37	-5.32 (-12.48,1.83)	0.13
Know my doctor did not receive payments	1.56 (0.56,2.56)	0.003**	2.23 (1.07,3.39)	0.02**	2.74 (1.28,4.20)	0.002**
**Wake Forest measure of trust in medical profession (5=lowest trust, 25=greatest trust)**						
Do not know if my doctor received payments	ref		ref		ref	
Know my doctor received payments	1.64 (0.50,2.78)	0.006**	2.10 (0.68,3.52)	0.009**	1.09 (-3.02,5.20)	0.57
Know my doctor did not receive payments	1.26 (0.33,2.20)	0.009**	2.06 (0.87,3.24)	0.004**	2.52 (0.44,4.59)	0.03*
**Expertise** **% agree or strongly agree: my doctor is a real expert in taking care of medical problems like mine**						
Do not know if my doctor received payments	ref		ref		ref	
Know my doctor received payments	-10.8% (-25.4%,3.8%)	0.14	2.5% (-6.2%,11.1%)	0.53	-5.5% (-1.9%,-0.0%)	0.042*
Know my doctor did not receive payments	8.2% (-1.3%,17.6%)	0.09	6.9% (-9.4%,23.2%)	0.36	23.6% (17.1%,30.1%)	<0.001**

© 2019 Kanter GP et al. *JAMA Network Open*.

Satisfaction with health care<br>% satisfied or very satisfied: in general, how satisfied are you with the health care you received in the past 12 months from your doctor?						
Do not know if my doctor received payments	ref		ref		ref	
Know my doctor received payments	-16.7%<br>(-28.1%,-5.3%)	0.005**	-17.3%<br>(-23.1%,-11.4%)	<0.001**	2.0%<br>(-30.0%,33.4%)	0.89
Know my doctor did not receive payments	4.1%<br>(-2.2%,10.3%)	0.19	2.6%<br>(-14.5%,20.0%)	0.73	10.4%<br>(-4.3%,25.1%)	0.15

** significant at 0.01 level *significant at 0.05 level

Notes:

a. All analyses are based on a balanced panel of individuals who did not change doctors between Wave 1 and Wave 2.

b. Additional covariates include age, gender, race/ethnicity, education categories, urban residence, household income categories, employment categories, previous diagnosis of chronic conditions (which include acid reflux, asthma, atrial fibrillation, COPD, chronic pain, cystic fibrosis, diabetes, epilepsy, eye disease, gout, heart disease, hepatitis C, hypertension, high cholesterol, HIV, kidney disease, multiple sclerosis, osteoarthritis, osteoporosis, rheumatoid arthritis, sleep disorder), previous diagnosis of cancer, previous diagnosis of stroke or myocardial infarction, previous diagnosis of mental health disorder, number of visits to the doctor, whether insured, quadratic terms of age and number of visits to account for non-linearities in age and number of visits. Standard errors were clustered at the state level.

© 2019 Kanter GP et al. *JAMA Network Open*.
